# A Fast and Accurate Mapping Method for an OPGW Tower Based on Hybrid Distributed Optical Fiber Sensing

**DOI:** 10.3390/s24175629

**Published:** 2024-08-30

**Authors:** Yuanyuan Yao, Ruofan Wang, Hao Ding, Shuai Tong, Yucheng Han, Shisong Zhao, Ningmu Zou, Fei Xiong, Yixin Zhang

**Affiliations:** 1 College of Engineering and Applied Sciences, Nanjing University, Nanjing 210023, China; dg20340012@smail.nju.edu.cn (Y.Y.); wrf@smail.nju.edu.cn (R.W.); 622022340003@smail.nju.edu.cn (H.D.); tongshuai@smail.nju.edu.cn (S.T.); 622023340007@smail.nju.edu.cn (S.Z.); nzou@nju.edu.cn (N.Z.); 2 Key Laboratory of Intelligent Optical Sensing and Manipulation, Ministry of Education, Nanjing University, Nanjing 210093, China; 3 Nanjing Fiber Photonics Technology Co., Ltd., Nanjing 211135, China; zyx@fib-tech.com; 4 Inner Mongolia DT Electric Power Design, Hohhot 010010, China

**Keywords:** hybrid DOFS, tower mapping, OPGW, dark fiber

## Abstract

The combination of the dark fiber in existing Optical Fiber Composite Overhead Ground Wire (OPGW) with Distributed Optical Fiber Sensing (DOFS) technology can be used to enable online monitoring and provide early warnings of anomalies in high-voltage transmission lines. Accurate mapping of the optical cable length to the geographic coordinates of actual towers is a key factor in achieving this goal. This paper discusses the principle of using a DOFS system for transmission line tower positioning and presents four available positioning features. To overcome the limitations of single physical parameter positioning, this paper presents a self-developed hybrid DOFS that simultaneously captures Rayleigh backscattering and Brillouin scattering signals. Several physical parameters, including temperature, strain, and vibration, are acquired synchronously. Through hybrid multi-parameter analysis, the rapid and accurate positioning of OPGW line towers is achieved. Experimental results have shown that the proposed method, based on the hybrid DOFS system, can locate up to 82 towers, while the traditional method could only identify 12. The hybrid system was able to complete 80% of the tension towers in 40 h. This paper presents a novel multi-parameter localization method that has the potential to significantly improve the efficiency and reliability of grid operation and maintenance.

## 1. Introduction

The power grid is an essential piece of infrastructure supporting national economies and social development. China has established the world’s largest power supply and clean power generation system [1]. In 2023, the length of new 220 kV and above transmission lines reached 38,100 km, while the transregional transmission of electricity amounted to 849.7 billion kilowatt-hours [2]. Nevertheless, power transmission lines are sensitive to a variety of natural and artificial factors, leading to detrimental effects such as icing [3], galloping [4], and lightning strikes [5,6]. The occurrence of such incidents may have significant economic and social implications. However, current research indicates that it is still challenging to achieve rapid and accurate fault location in the event of a transmission line fault [7,8].

Optical Fiber Composite Overhead Ground Wire (OPGW) is a highly reliable and cost-effective solution for high-voltage overhead transmission lines [9]. This technology combines the original electrical and mechanical properties of overhead ground wires with the communication function of optical fiber cables [10]. Distributed optical fiber sensor (DOFS) technology employs optical fibers as both sensing and transmitting media, facilitating the monitoring of temperature, vibration, strain, and other physical parameters along the fiber [11]. By combining DOFS technology with dark fiber in OPGW, it is possible to effectively obtain sensing ability for power transmission lines with minimal modification, enabling long-term and uninterrupted online monitoring.

Existing DOFS applications in transmission line monitoring focus on monitoring anomalies such as icing [12,13,14,15], galloping [16,17,18], and lightning strikes [19,20]. However, accurate mapping between the length of the sensing optical fiber cable and the actual geographic coordinates of the power transmission line must also be resolved for this technology to be applied in the field. During the design and construction of transmission lines, each tower is assigned a unique number, and its geographic coordinates are clearly defined in terms of latitude, longitude, and height. In order to prevent external stress from damaging the fiber, it is essential that the internal fiber remains relaxed. Consequently, OPGW cables are manufactured with a certain residual length of internal fiber compared to the external cable [21]. The OPGW cables suspended between the towers exhibit a certain curvature, such that the physical distance between the towers does not directly correspond to the fiber span length. Furthermore, even after transmission line construction has been completed, a number of factors may cause additional shifts in the position of the OPGW cables relative to the tower, leading to inaccuracies in the location data as the transmission line lengthens. This complicates the task of guiding operations and maintenance personnel in locating anomalies in the field. As a result, a tower mapping method is essential to establish the correlation between optical fiber length and actual geographic coordinates.

DOFS technology is capable of monitoring differences in temperature, strain, vibration, and other characteristics when locating towers, and the entire line can be mapped using interpolation and other methods. In 2018, Xie et al. [22] employed a phase-sensitive optical time-domain reflectometer with a spatial resolution of 3.4 m to monitor variations in dynamic strain along the transmission line during artificially stimulated galloping. The results indicate that the strain amplitude at the tower location is considerably less than that of the suspension section. This observation suggests that the latter may be employed in finding the location of the tower. However, this method has not yet been verified in field experiments. In 2017, Huang et al. [23] employed Brillouin optical time domain reflectometry to map a 500 kV transmission line tower. They utilized the difference in the average Brillouin frequency shift (BFS) caused by spliced optical fibers in the OPGW line and the smaller variance in BFS at the splice closure to locate the towers. Nevertheless, the monitoring data were selected based on the results from large temperature differences on sunny days, which did not allow for the possibility of positioning quickly around the clock. In 2021, Xia et al. [24] explored a theory on the BFS jump phenomenon at the optical fiber splicing point. The results of the simulations indicate that fibers with different initial BFSs will exhibit the BFS jumping phenomenon on both sides of the fiber fusion point. Taking into account this research, they proposed a methodology for combining the results of multiple evaluations of fiber cores. In 2022, Li et al. [25] proposed the application of the DBSCAN algorithm for the rapid verification of step points in BFS curves, with the objective of achieving the precise positioning of splice closures. Despite its efficacy, the complex nature of the algorithm has limited its ability to effectively position all towers.

The above work mainly focuses on towers equipped with splice closures, utilizing the BFS jump phenomenon. It is crucial to clarify that the proportion of these unique towers in power transmission lines is relatively small, and this is not good enough to achieve uniform accuracy all along the power transmission line. Furthermore, the accuracy and speed of existing single parameters are significantly affected by meteorological conditions in the vicinity of the transmission line. Therefore, a hybrid distributed optical fiber sensing (H-DOFS) system was introduced to monitor transmission lines using multiple parameters simultaneously by capturing Rayleigh backscattering (RBS) signals and Brillouin scattering signals. We put forth four categories of tower mapping criteria that encompass all varieties of towers and propose the implementation of an interpolation methodology to complete missing towers and accurately localize anomalous events. Experimental validation is conducted in a field setting to achieve the rapid, precise, comprehensive, and universally applicable positioning of transmission line towers.

## 2. Principle of Transmission Line Tower Mapping Based on DOFS

### 2.1. High-Voltage Overhead Transmission Line Tower Types and Structures

**Figure 1:**
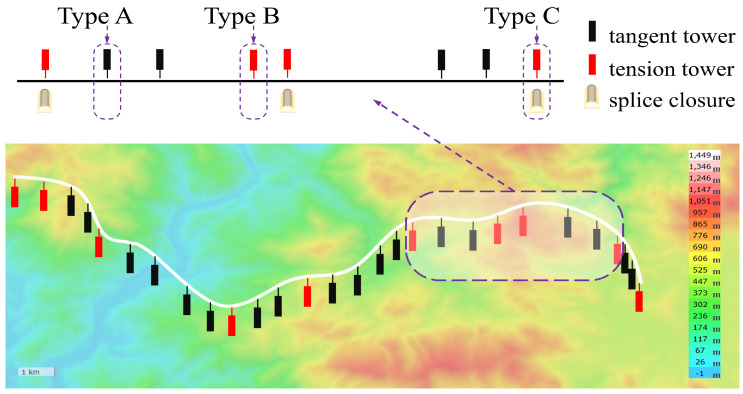
Schematic diagram of high-voltage overhead transmission tower-line system.

A typical high-voltage overhead transmission line tower system is shown in Figure 1. The design and construction of the transmission line include the installation of tension towers and tangent towers according to the geographical environment and transmission needs [26]. Tangent towers support the OPGW and accessories with a suspended structure, which allows moderate tower deflection, improving its adaptability to the environment. Tension towers carry the strain along the line direction, the vertical load of the transmission line, and the wind load in the horizontal direction.

In transmission lines, fiber optic cable splicing is necessary because of the limits of the length of a single fiber optic cable. Splicing is typically conducted on a tension tower. Consequently, transmission line towers are classified into three categories: A-type and B-type are tangent and tension towers, respectively, and C-type is a tension tower with a splice closure, as illustrated in Figure 1.

### 2.2. Principle of Tower Mapping

#### 2.2.1. Tower Mapping Based on BFS Jump

**Figure 2:**
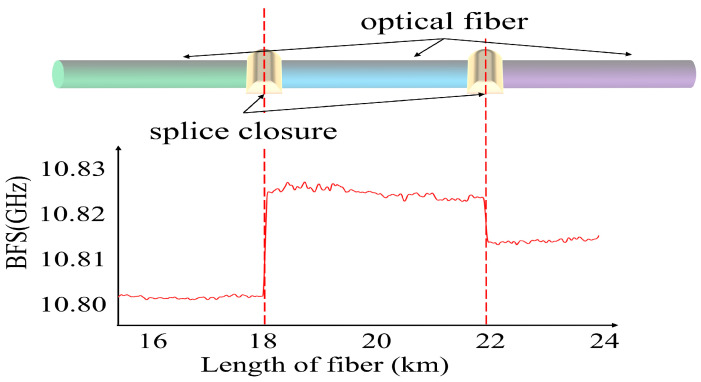
BFS jump at the optical fiber splicing point.

In high-voltage overhead transmission lines, optical fiber cables exhibit varying initial BFSs due to differences in fiber materials, manufacturing processes, manufacturers, and batches [16]. If there is a difference in the initial BFS of optical fibers on both sides of a fusion splice at the tower, a BFS jump may occur at the splice point, as shown in Figure 2. Therefore, the traditional method of tower positioning relies on detecting BFS discontinuities to locate fiber joint closures accurately, thereby anchoring the positions of C-type towers effectively. However, C-type towers make up a relatively small percentage of the line, resulting in insufficient locating anchor points, which subsequently leads to lower mapping accuracy.

#### 2.2.2. Tower Mapping Based on Vibration Amplitude Differences

**Figure 3:**
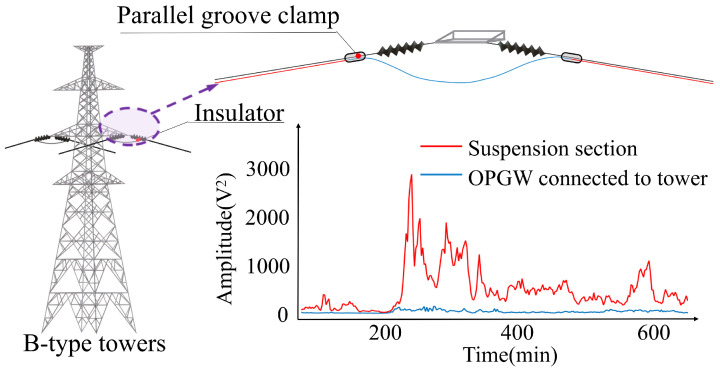
Schematic diagram of the structure at OPGW through B-type towers with amplitude reduction phenomenon.

Overhead transmission lines are affected by environmental factors such as galloping, which causes varying cable vibration amplitudes. The cables are restricted at the tower, limiting their ability to vibrate freely and causing some vibration energy to be absorbed at that location. B-type towers serve to carry the line tension, exert a stronger stabilizing effect on the connected cable segments, and thus result in a significantly lower vibration amplitude of the connected conductor segments than that of the free conductor segments, as shown in Figure 3. To prevent the deterioration of the outer armor of the optical fiber cable, the OPGW down-lead and the remaining cable are affixed to the tower with a stationary fixture. The fixed cable section is typically greater than 50 m in length [27]. As a result, the vibration amplitude can be significantly reduced, as can be easily observed in the case of C-type towers. The exact location of B-type towers and C-type towers in the OPGW line can be determined. This positioning method identifies towers without relying on optical fiber splice points, providing greater universality and reliability.

#### 2.2.3. Tower Mapping Based on Temperature Differences

**Figure 4:**
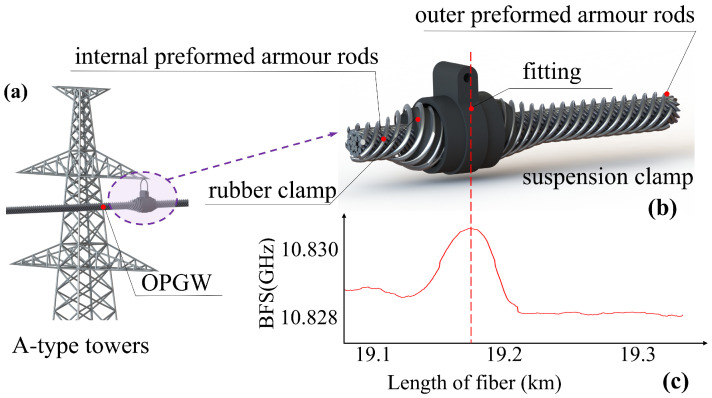
Schematic diagram of OPGW straight-line tangent tower structure. (**a**) Straight-line tangent towers. (**b**) The suspension clamp. (**c**) The temperature rise phenomenon.

A-type towers support OPGW cables hanging from suspension clamps, as shown in Figure 4a. Figure 4b illustrates the specific structure of the suspension wire clamp.

In sunny weather, the black rubber absorbs more heat under direct sunlight, causing the local area to heat up more quickly and often resulting in higher temperatures than the surrounding area. The internal structure of the OPGW cable consists of optical fibers housed in aluminum alloy tubes. The cavities are filled with insulating grease to prevent the localized temperature increase from spreading rapidly to the surrounding metal conductors, which would result in the occurrence of temperature spikes, as shown in Figure 4c. By analyzing these temperature anomalies caused by solar radiation, A-type towers can be located quickly and accurately.

#### 2.2.4. Tower Mapping Based on Strain Differences

**Figure 5:**
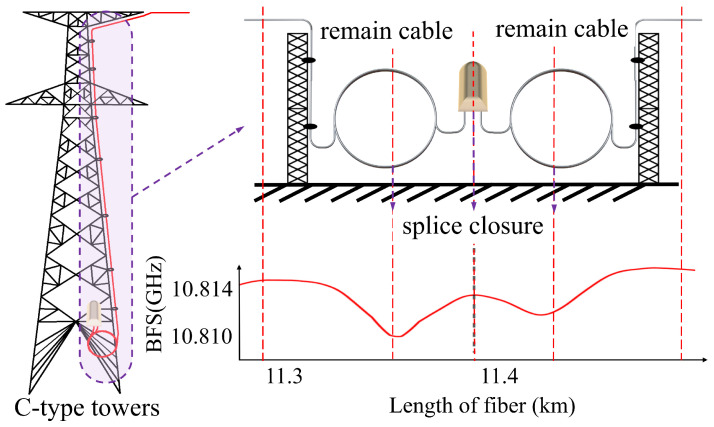
Strain characteristics of C-type towers.

The internal stresses in OPGW cables are a consequence of the thermal expansion and contraction of the materials due to changes in the ambient temperature. Suspended optical fiber cables are subject to both their own weight and the horizontal tension resulting from line sag, which leads to a significant increase in internal stresses as temperatures rise. In contrast, the remaining cables attached to the tower do not carry their own weight, resulting in minimal tension on the internal fibers. As ambient temperatures rise, differential heating between the interior and exterior of the splice closure, coupled with the disparate rates of thermal expansion, gives rise to localized stress. These conditions are manifested in the form of typical “W-type” stress localization features observed on splicing towers, as illustrated in Figure 5. By monitoring the stress changes along the OPGW cable and capturing this feature, it is possible to identify C-type towers.

**Figure 6:**
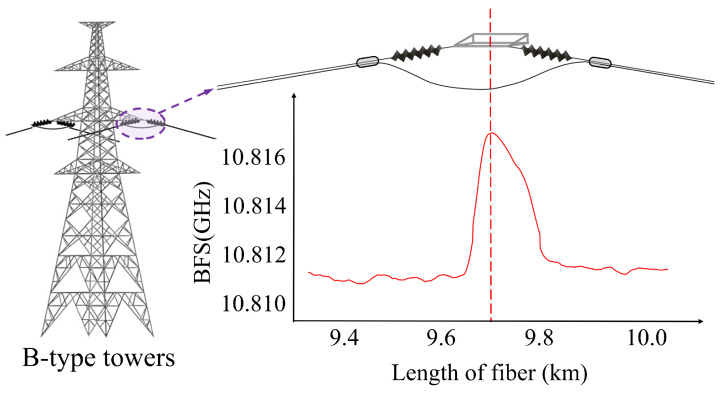
Strain characteristics of B-type towers.

In addition, B-type towers without fiber splicing use jumper cables for electrical isolation. As the ambient temperature rises, the optical fiber cable undergoes thermal expansion and contraction, increasing the internal stress at the junction points with the insulator strand. This often appears as a single, wider stress peak due to spatial resolution limitations, as illustrated in Figure 6. Capturing this stress peak feature allows for the identification of B-type towers.

The four tower location methods described, each relying on a single parameter and feature, can be used to effectively locate various transmission line towers under different conditions. However, each method has its limitations, particularly in the limited number of towers they can accurately locate when used independently. Additionally, the temperature difference method and the strain difference method are more sensitive to environmental influences. To address these challenges, we propose H-DOFS, which combines the four positioning methods by monitoring the line vibration, temperature, stress, and other multi-parameters simultaneously. This approach aims to overcome the constraints of individual positioning methods and enhance the comprehensive, rapid, and accurate positioning of the transmission line towers.

## 3. Tower Mapping Method Based on H-DOFS

### 3.1. Realization of the H-DOFS System

In this system, the phase variation in the RBS is employed as a carrier for the vibration signal. Brillouin scattering is employed as the carrier of the strain and temperature coefficients in order to align the sensing distance and signal strength of the vibration sensing. In the H-DOFS, the distribution of backward Rayleigh-scattered optical power, Brillouin-scattered optical power, and Brillouin-scattered optical frequency shift in the optical fiber are obtained simultaneously.

**Figure 7:**
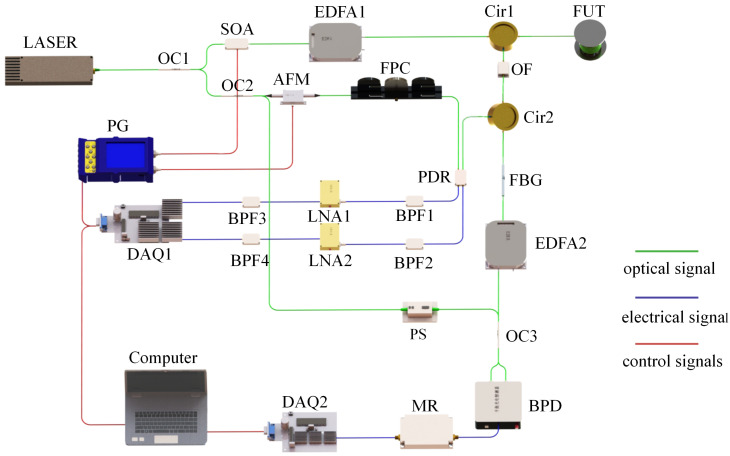
Structure of the H-DOFS system.

The structure of the sensing system is depicted in Figure 7. The system employs the narrow linewidth laser as the light source and divides the light wave into two paths through a 90:10 coupler (OC1). A pulse-modulated semiconductor optical amplifier (SOA) modulates 90% of the light into pulsed light, which is then amplified by erbium-doped fiber amplifiers (EDFA1) to form the optical probe pulse. This light enters the fiber under test (FUT) through the circulator (Cir1) to perform the detection. The backscattered light of the FUT returns from port3 of Cir1 and is filtered out by an optical filter (OF) with a bandwidth of 0.8 nm to remove the broadband amplifier’s spontaneous emission noise. It then enters the fiber Bragg gratings (FBG) through Cir2 to separate the RBS signals from the polarization diversity receiver module for processing. Meanwhile, the FBG outputs the Brillouin scattering signals in the backscattered light signal to the EDFA2 for amplification.

In the reference optical path, 10% of the light is divided into two paths, with one path comprising 50% of the total. The OC2 is employed to direct a single path through an acousto-optic frequency shifter (AFM) in order to generate a 200 MHz frequency-shifted intrinsically oscillating light. This light is then adjusted to the polarization state through a fiber polarization controller (FPC) before being coherently combined with the RBS signals to generate the intermediate frequency signals. These signals are then processed through a band-pass filter (BPF) with a center frequency of 200 MHz and a low-noise amplifier (LNA) before being acquired during data acquisition (DAQ1). The second path involves the Brillouin scattering light of the principal oscillator, which is scrambled by the polarization scrambler (PS) to randomize its polarization state. This process results in the generation of a beat frequency, which is derived from the amplified Brillouin scattering signal of EDFA2. This signal is then subjected to mixing and power detection by the microwave receiver (MR) and synchronously acquired by the high-speed DAQ2 for subsequent data processing. The parameters of the H-DOFS system used in this paper are shown in Table 1.

Due to the very weak Brillouin scattering signal, multi-point averaging is required and the number of averages will depend on the local climate conditions. Consequently, during different periods of temperature and strain measurement, single measurement time will vary, ranging from 40 min to 2 h, resulting in uneven intervals along the time axis. However, even with uneven sampling, we can still discover and extract the intrinsic regularity of changes in these parameters over time from the collected data.

Because of the linear relationship between frequency shift and power change in Brillouin scattered light with variations in temperature and stress [28], and the sensitivity of Rayleigh scattering phase changes to fluctuations in temperature and strain [29], a set of superdetermined equations can be constructed by combining the sensitivity characteristics of Rayleigh and Brillouin scattering to temperature and strain.

(1) $$\left\{\begin{array}{c} \Delta v_{B}=C_{v\varepsilon }\Delta \varepsilon +C_{vT}\Delta T\\ \frac{\Delta P_{B}}{P_{B}}=C_{P\varepsilon }\Delta \varepsilon +C_{PT}\Delta T\\ \Delta \phi =\left(1+C_{\varepsilon }\right)nkLe_{z}\\ \Delta \phi =\left(\xi +C_{T}\right)n_{eff}kL\Delta T \end{array}\right.$$

In this context, Δε and ΔT represent the stress change and temperature change, respectively. Similarly, $\Delta v_{B}$ denotes the Brillouin frequency shift change, while $\Delta P_{B}$ signifies the Brillouin power change. Δϕ corresponds to the phase shift of the optical fiber. Additionally, $C_{v\varepsilon }$ and $C_{vT}$ are the strain coefficient and temperature coefficient of the Brillouin frequency shift, respectively, while $C_{P\varepsilon }$ and $C_{PT}$ are the strain coefficient and temperature coefficient of the Brillouin power, respectively. ξ represents the thermal expansion coefficient of the optical fiber, and $n_{eff}$ stands for the equivalent refractive index. $C_{T}$ represents the thermo-optical coefficient, and $C_{\varepsilon }$ signifies the elastic optical coefficient. In order to obtain the above coefficients, a pre-calibration using the same OPGW is required. By using the superdetermined equation and the sensitivity of the Rayleigh scattering effect to vibration, it is expected that the cross-modulation decoupling of vibration, strain, and temperature can be realized, which makes the synchronous and high-precision monitoring of multi-parameters possible.

### 3.2. Tower Mapping for the Entire Transmission Line

**Figure 8:**
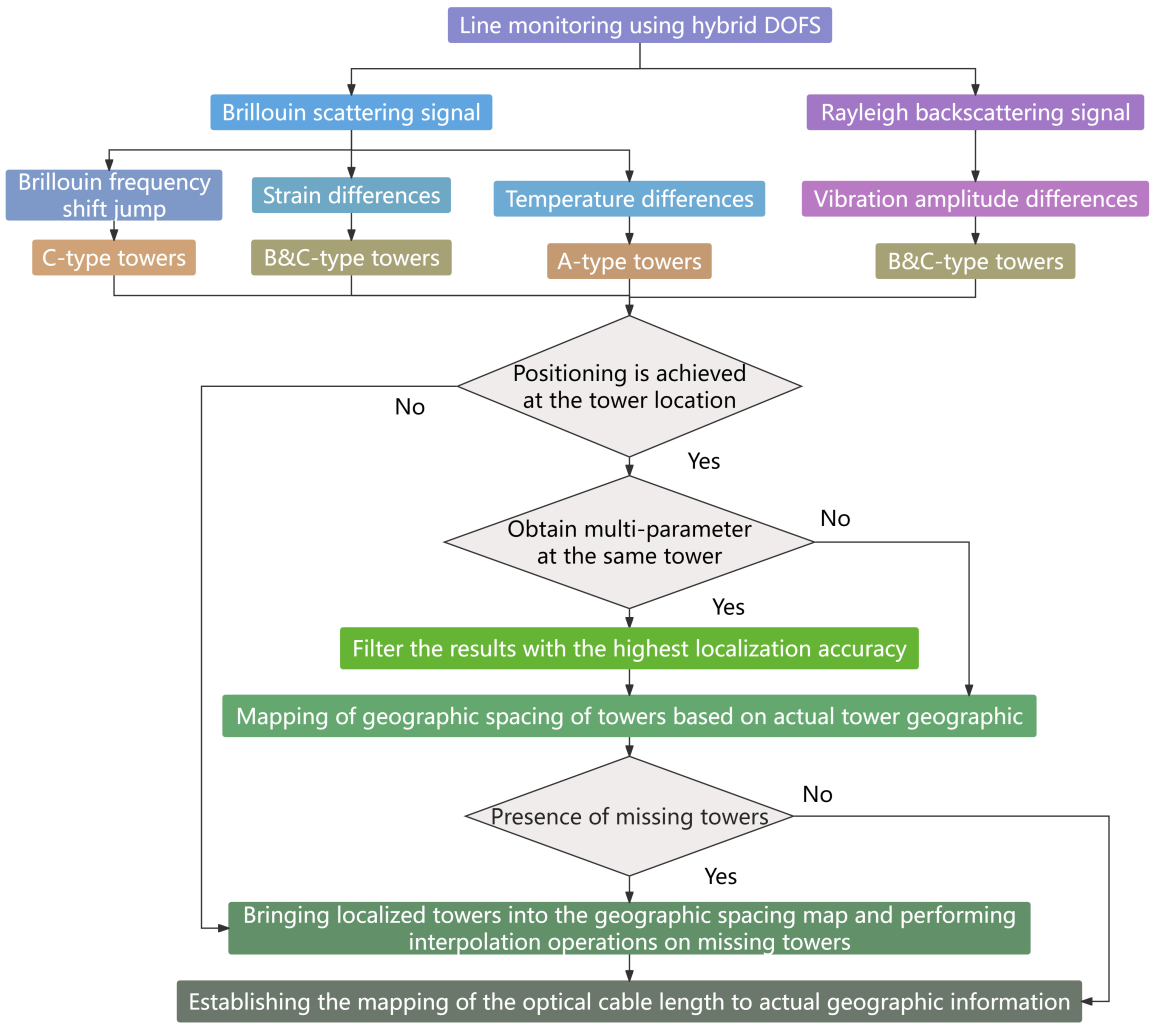
Tower mapping process flow chart.

The process flow chart to achieve accurate positioning of the monitoring events using tower mapping is shown in Figure 8. The Brillouin scattering signal and Rayleigh backscattering signal collected by the H-DOFS can be utilized to monitor the BFS, strain, temperature, and vibration of the line after signal processing. The most accurate data are prioritized for preliminary positioning results.

In high-voltage overhead transmission lines, there is generally a linear proportional relationship between the optical cable length and the actual geographic location. When performing calculations for missing towers and establishing mapping relationships, the additional fiber length introduced by the installation structure at the C-TYPE tower can be considered negligible. The error resulting from the arc drop of the overhanging section and the remaining fiber length can be approximated as a linear proportional relationship, which allows the location of the missing tower to be determined using a linear interpolation algorithm. Once the entire line has been accurately mapped, further confirmation of the exact location of an anomaly within a specific span would be required. In this situation, the relationship between the overhead cable and the actual geographic location can no longer be approximated linearly. It is essential to consider the arc droop function of the overhead line for accurate location interpolation.

If a tower position is missing, the formula for the interpolation operation is given by

(2) Y=F(l)

where *Y* and *l* represent the location of the fiber length and the exact geographic coordinates. The function *F* is a differential function influenced by the environment and the line parameters.

When calculating the position of missing towers using the interpolation algorithm, *F* is a linear interpolation function. Given known geographic distances $l_{1},l_{2}\ldots l_{n},l_{n+1},l_{n+2}\ldots$ and corresponding tower positions $Y_{1},Y_{2}\ldots Y_{n},Y_{n+1},Y_{n+2}\ldots$ from the H-DOFS, if the geographic coordinates $l_{n}$ correspond to the tower position $Y_{n}$, and $l_{n+2}$ corresponds to $Y_{n+2}$, then for the unlocated tower N+1, the position $Y_{n+1}$ is interpolated using the known points $\left(l_{n},Y_{n}\right)$ and $\left(l_{n+2},Y_{n+2}\right)$.

Calculate the slope *m* as follows:

(3) $$m=\frac{Y_{n+2}-Y_{n}}{l_{n+2}-l_{n}}$$

Then, the value of $Y_{n+1}$ is

(4) $$Y_{n+1}=Y_{n}+m\left(l_{n+1}-l_{n}\right)$$

Once accurate positioning of the line towers has been achieved, a mapping relationship can be established between the optical cable length of the entire line and its actual geographic coordinates. When H-DOFS detects an abnormal event, it is first localized approximately to the interval between two towers according to the established mapping relationship. Subsequently, the interpolation algorithm is employed to ascertain the precise geographic coordinates of the event within the specified interval. In this context, the *F* function refers specifically to the sag of the line.

The state equation for the length of the overhead line is established according to the hanging chain line method [30], as follows:

(5) $$S=\int_{0}^{x}\sqrt{1+{y^{\prime }}^{2}}dx=\frac{T_{0}\cos\varphi }{q}\left[\sinh\left(\frac{q}{T_{0}\cos\varphi }x+k\right)-\sinh k\right]$$

**Figure 9:**
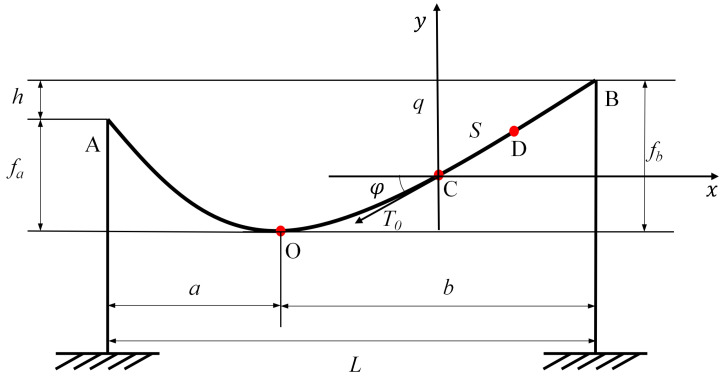
Force diagram of the overhead line.

The force diagram of the overhead line is shown in Figure 9, where *S* is the length of the catenary in the CD section, $T_{0}$ is the tensile force at the left endpoint *C*, φ is the angle between the tangent direction at endpoint *C* and the horizontal, *h* is the difference in horizontal heights of point *A* and point *B*, and *q* is the dead weight per unit length of the overhead line. *x* represents the horizontal distance from point C to any point on the curve, and $y^{\prime }$ denotes the slope of the curve at that point. *k* is defined in Equation (6).

(6) $$k=\mathrm{arcosh}\frac{1}{\cos\varphi }$$

Therefore, the total length of the AB section of the overhead line is the sum of the length of the AO section and the length of the OB section, which is obtained by integrating Equation (7):

(7) $$L_{AB}=T_{0}\cos\varphi \left[-\sinh\left(a\right)+\sinh\left(\frac{a\cdot q}{T_{0}\cos\varphi }\right)\right]+T_{0}\cos\varphi \left[-\sinh\left(b\right)+\sinh\left(\frac{b\cdot q}{T_{0}\cos\varphi }\right)\right]$$

where *a* represents the horizontal distances from the fixed point *A* on the left to the origin *O*, and *b* represents the horizontal distances from the origin *O* to the fixed point *B* on the right. Accordingly, the actual geographical location of the event can be calculated. Taking an event occurring at a distance $S_{0}$ from the tower location as an example, $S_{0}$ is first compared with $L_{AB}$ and $L_{AO}$, calculated in Equation (7), to confirm its location in the zone, and then its actual geographic coordinates *D* are calculated:

(8) $$D=\frac{T_{0}\cos\varphi }{q}\times \mathrm{arsinh}\left[\frac{S_{0}}{T_{0}\cos\varphi }+\sinh\left(k\right)\right]-k$$

## 4. In-Field Test and Experimental Results Analysis

From January to March 2023, our group utilized a H-DOFS to monitor a 220 kV line in Fujian, PRC. The H-DOFS is installed in the communications room at one end of the OPGW line. Tower positioning was accomplished by analyzing data from the H-DOFS. The spatial resolution for monitoring RBS signals was set at 50 m to cover the dynamic range requirement for the overall length of the monitoring section. The 220 kV line has a total length of 44.16 km and includes 61 A-type towers, 29 B-type towers, and 12 C-type towers. Its largest segment is up to 1016 m. The type and number of towers that can be localized by a single parameter are shown in Table 2.

**Figure 10:**
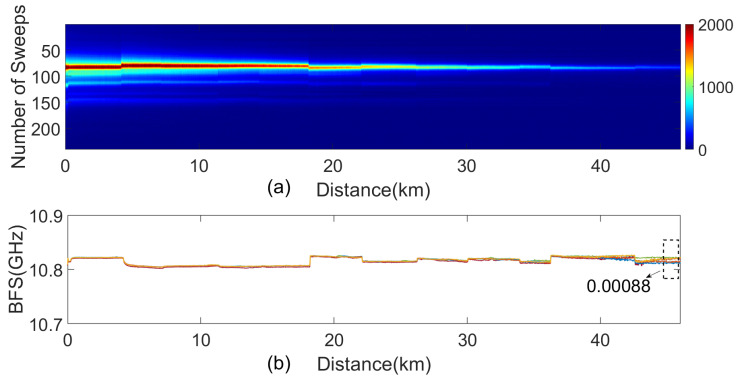
The actual measured Brillouin scattering signal. (**a**) Spatial distribution of Brillouin scattering power spectra. (**b**) Brillouin frequency spectrum signal.

The actual measured spatial distribution of Brillouin scattering power spectra is shown in Figure 10a. Figure 10b shows the continuous Brillouin spectrum signals from adjacent time periods. As seen from the figure, the overall fluctuation is minimal. The calculated BFS error at the 45–46 km segment of OPGW has an average standard deviation of 0.88 MHz, which meets the experimental requirements.

**Figure 11:**
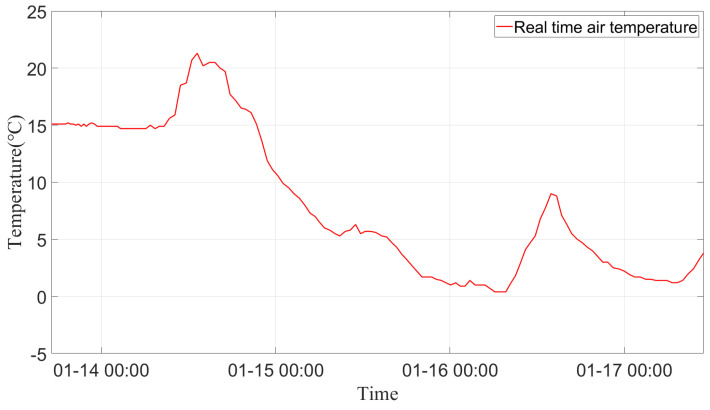
Real-time air temperature at the test site, 14 January 2023–17 January 2023.

Figure 11 shows real-time temperature changes from 14 to 17 January 2023, as recorded by the line sensors. The 14 January 2023 marked a sunny, warm day after a period of low temperatures, providing valuable data to determine tower locations based on temperature and strain differences. As temperatures increase, the specific structure of the tower may induce both stress and temperature changes, with temperature changes being the primary factor.

### 4.1. Identification of Towers

#### 4.1.1. Brillouin Scattering Signal Processing

**Figure 12:**
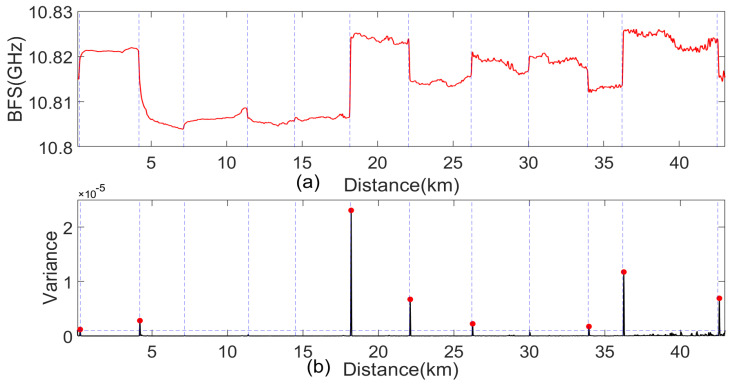
Results of identification using BFS. (**a**) BFS curve. (**b**) BFS sliding window variance and jump determination results.

The Brillouin scattering signals collected by the DOFS were first denoised and then subjected to a curve fit [31] to obtain the BFS curve, as shown in Figure 12a. The BFS curves were analyzed using a sliding window length variance calculation method. The design principle of the sliding window size is as follows: the sliding window size should be slightly larger than the total length of the lead wires and residual cables at the single connection tower and smaller than the distance of any section of the measurement line. This ensures that each window includes only one tower. Regions where the variance exceeds the average variance across all sliding windows of the BFS curve were identified as potentially anomalous regions. For each potentially anomalous region identified, anomalies in the BFS were further localized using local peak detection. The lines monitored exhibit an average stall spacing of 432 m, tower heights of approximately 30–50 m, and caliper heights of 18–30 m. Consequently, the sliding window size was set to 60 m. The variance data were analyzed using a peak-finding algorithm to precisely mark the anomaly locations, as shown in Figure 12b. The algorithm identified a total of eight BFS jump points in all twelve C-type towers along the line.

#### 4.1.2. Rayleigh Backscattering Signal Processing

**Figure 13:**
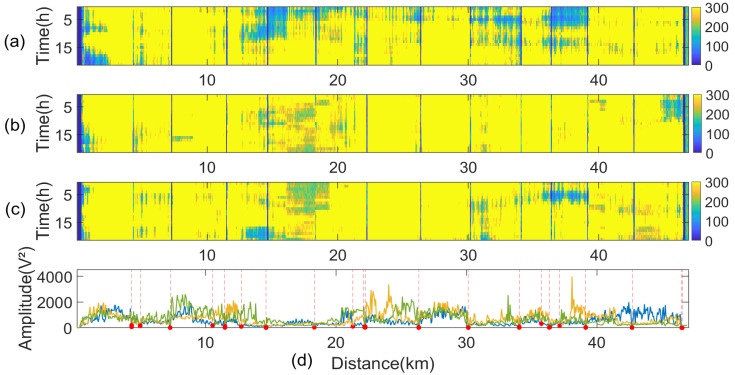
Results of identification using amplitude. (**a**) Short-time energy diagram of 0–20 h vibration signal. (**b**) Short-time energy diagram of 20–40 h vibration signal. (**c**) Short-time energy diagram of 40–60 h vibration signal. (**d**) Energy statistics for each respective time period.

Figure 13a–c display the spatial–temporal distribution maps of vibration signals obtained at different times, while Figure 13d shows the two-minute short-term energy statistics for each respective time period.

The short-term energy map of the vibration signal was analyzed using a sliding window variance calculation method to identify regions of significantly reduced amplitudes. Areas where the variance values fall below a predetermined threshold were flagged as potential anomalies, indicating possible pylon locations. De-emphasis was performed on potential towers to eliminate duplicate determinations that may be caused by environmental changes or the down-lead on the towers. Finally, the point with the lowest vibration energy among the neighboring points was selected as the exact location of the towers. Due to the influence of environmental factors such as galloping on the amplitude of overhead transmission line cable vibrations, the position of the dark stripes may vary. After multiple measurements, an overall recognition can be achieved.

During data processing, the sliding window size and threshold settings should be adjusted to the specific conditions of the monitored line. The design of the sliding window should consider the overall length of the line, the number of tension towers, and the spatial resolution of the DOFS. The fundamental principle is that the window length is slightly shorter than the average tension section length of the line, such that only one tension tower is included in a sliding window. Threshold settings should account for the amplitude differences in vibration between the suspended and fixed optical fiber cables at the tension towers, as well as the lengths of the down-lead and remaining cables. The mean stall spacing of the monitored lines is 432 m, and 40% of all towers are tension towers. Therefore, the size of the designed sliding window is set to 1 km, and the threshold is defined as one-sixth of the mean variance of the sliding window.

### 4.2. Speed and Accuracy of Tower Mapping

#### 4.2.1. The Speed of Tower Mapping in a Single Parameter and Hybrid System

**Figure 14:**
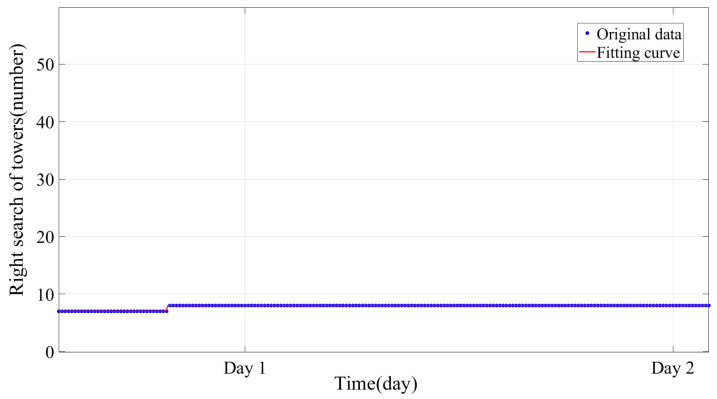
Mapping results of BFS jump.

**Figure 15:**
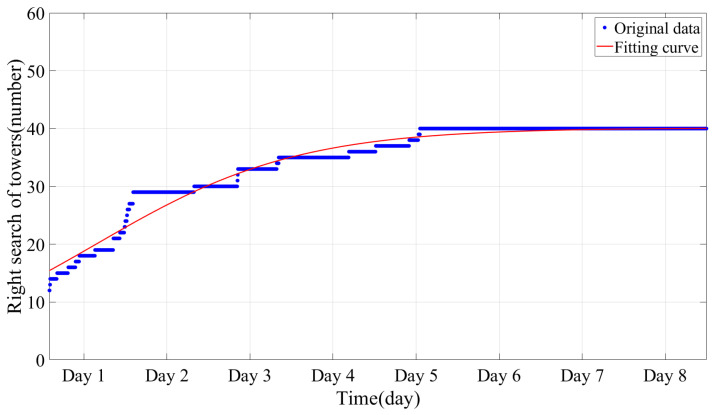
Mapping results of vibration amplitude differences.

**Figure 16:**
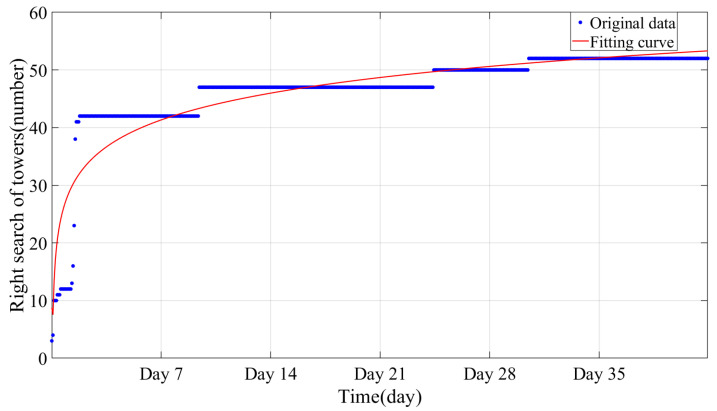
Mapping results of temperature difference.

**Figure 17:**
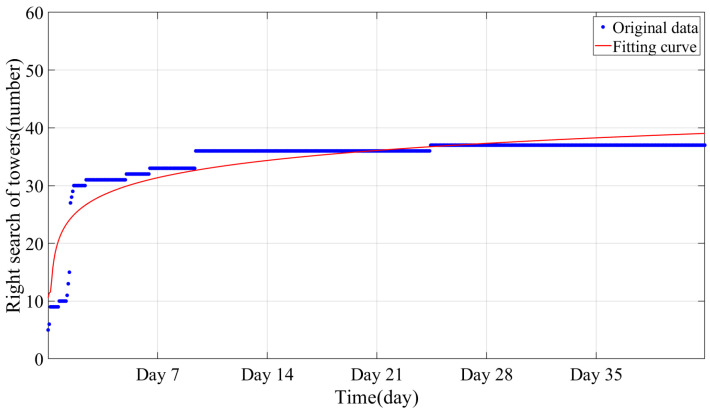
Mapping results of strain difference.

Figure 14, Figure 15, Figure 16 and Figure 17 show the monitoring results, illustrating the four previously mentioned tower positioning characteristics using the H-DOFS. The blue solid points represent the raw data, while the red realizations represent the fitted curves.

Figure 14 shows the monitoring determination using the BFS jump for C-type towers. It shows that the majority of towers are identified early in the first location, with a single set of monitoring data able to pinpoint seven out of the twelve C-type towers. However, the identification of this method shows a slow growth with increasing monitoring time. After 14 h, only one new tower had been located. In the end, the method identified a total of eight towers. The fitting function for this method is a step function, indicating that although the method can locate quickly, the location rate is limited.

Monitoring determinations for the locations of B- and C-type towers are shown in Figure 15. With the accumulation of time, the location rate had an obvious improvement. After 54 h, 80% of the 41 towers had been accurately located, with this figure rising to 90% after 94 h. The final positions of 40 towers were located, including 12 C-type towers and 28 B-type towers, with recognition rates of 100% and 96.5%, respectively. The logistic function model indicates a growth pattern that gradually reaches a plateau as more towers are identified. As the environmental wind around the line fluctuates, the number of B- and C-type towers that can be accurately determined increases gradually. Once the majority of these towers have been identified, the upper limit of the algorithm is considered to be reached.

Figure 16 shows the monitoring judgments using temperature differences to locate A-type towers. It shows a clear correlation between temperature and identification, with a notable increase during periods of sunny weather. The first day of observation, 14 January 2023, was marked by sunny, warm conditions following a period of low temperatures. In such instances, the occurrence of sustained low temperatures in conjunction with sunny weather can result in a rapid increase in the number of localized A-type towers identified by the temperature difference feature. Conversely, the rate of increase is comparatively slower during other times. The overall growth rate showed a stepwise rise. After 23 days, 80% of the 61 tangent towers were accurately located. However, this criterion is not effective for determining the number of A-type towers in situations where meteorological environmental conditions around the line do not meet the requisite criteria.

As illustrated in Figure 17, the monitoring results based on strain differences for the positioning of B- and C-type towers began on 14 January 2023. On that morning, a significant increase in identification was observed due to environmental influences. Subsequently, the growth rate exhibited slow and stable characteristics. After a monitoring period of nearly 8 days, 80% of the B- and C-type towers had been precisely located. This performance matches the accuracy achieved through pole tower positioning based on temperature differences.

**Figure 18:**
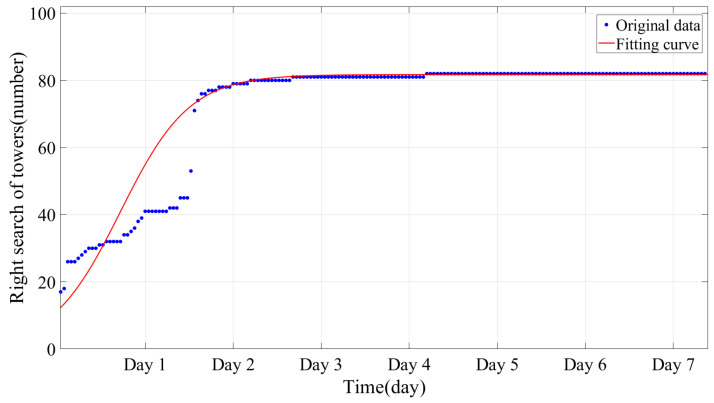
Mapping results of H-DOFS.

The total results obtained using H-DOFS monitoring are shown in Figure 18. During the initial two-day period, the identification rate of this method demonstrated a notable increase. A total of 78 towers were identified in 40 h, representing a significant increase from the initial 17 towers. The growth rate then slowed down. After 7 days of continuous monitoring, the H-DOFS successfully identified 42 A-type towers, 28 B-type towers, and 12 C-type towers, representing 68.8%, 96.5%, and 100% of all towers, respectively.

**Figure 19:**
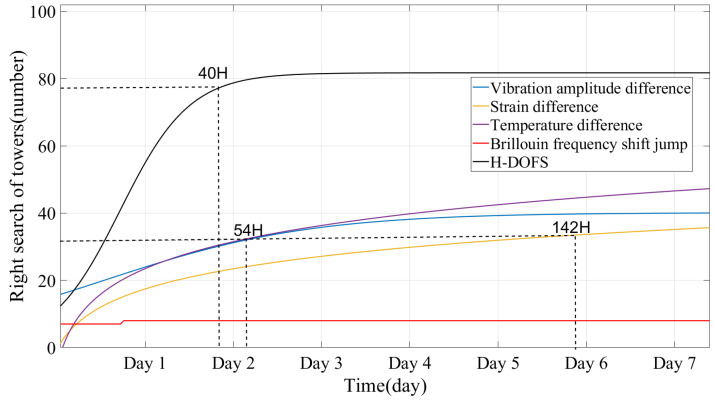
Statistics of tower determination results.

Figure 19 shows the statistics of the fitted curves derived from these monitoring results and H-DOFS. During the monitoring process, the tower identification rate of the H-DOFS consistently outperformed the single-parameter monitoring methods. In 40 h, the H-DOFS identified 37 B- and C-type towers and 41 A-type towers. This compares favorably to the 30 B- and C-type towers identified with a single strain parameter, 27 with a single amplitude parameter, and 8 with a single BFS jump. It represents a significant improvement in both the number and type of towers located. Logistic function fitting of these results indicates a significant influence of amplitude difference and BFS jump criteria over the monitoring period. The method demonstrates an 80% determination of B- and C-type towers in a mere 40 h, a markedly accelerated pace that is 0.35 and 6.1 times faster, respectively, than the 54 h required for a single amplitude parameter and 142 h for a single strain parameter.

#### 4.2.2. The Accuracy of Tower Mapping in a Single-Parameter and Hybrid System

To evaluate the accuracy of the position of the tower, the mean absolute error (MAE) between the actual and predicted positions of the tension towers over multiple measures is used as the primary index. The MAE is calculated using Equation (9):

(9) $$\mathrm{MAE}=\frac{1}{n}\sum_{i=1}^{n}\left|v_{i}-v\right|$$

where *n* is the number of measurements, $v_{i}$ is the *i*-th measurement, and *v* is the true value.

**Figure 20:**
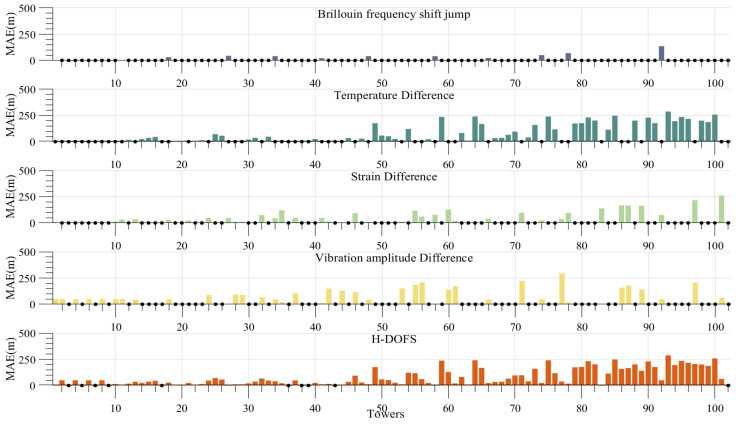
Interpolation calculation of positioning error.

Figure 20 illustrates the mean absolute localization error. The BFS jump characteristics for C-type tower positioning result in an average positioning error of 65 m after interpolation to account for the absence of towers. With regard to the disparity in vibration amplitude, the difference is 119 m. The average positioning error for the entire line is 124 m when the temperature difference characteristics are utilized to position the A-type towers. Taking into account the differential strain characteristics that distinguish towers of type B and C, the value in question is 88 m. Once the interpolation process has been completed, the average positioning error for the entire tower line using the H-DOFS is 84 m.

The relatively low overall positioning accuracy is primarily due to the low spatial resolution of Rayleigh scattering monitoring in the H-DOFS, which is further compounded by the long length of the line monitored in this experiment. A high-frequency omission can be observed in roughly the first third of the line. Upon analyzing the actual geographical location of the line, it can be seen that the reason for this phenomenon is that the starting section of the line is close to cities, where the temperature difference between day and night fluctuates less due to the activities of the residents, making it more difficult to use the characteristics of warming and clearing weather after prolonged low temperatures for the determination of A-type towers.

A comparison of the results of tower mapping for different mechanisms in seven days is presented in Table 3. The traditional tower positioning method relies on BFS jump detection, which is unaffected by weather and provides the highest precision in tower measurement. However, it can only locate up to 8 C-type towers, leading to insufficient anchor points. Even when combined with strain for joint positioning, the method is limited by the number of C-type towers, allowing for the location of up to 12 towers at most. The vibration amplitude difference method for tower mapping effectively determines B- and C-type towers with relatively high speed. Nevertheless, its precision is limited by the inherent constraints between spatial resolution and monitoring length. Tower mapping using temperature and stress differences is particularly adept in covering a wide area and achieving high levels of accuracy in tower mapping, but it is sensitive to environmental conditions, which can introduce some instability in the results. The H-DOFS is a novel approach that leverages the strengths of existing single-parameter methods. It can be employed to achieve the rapid and precise mapping of all types of towers along the entire line.

This paper proposes a multi-parameter fusion positioning method based on a hybrid system. By using the H-DOFS to obtain multiple physical parameters simultaneously and considering the structural characteristics of different towers, four categories are established to analyze the mapping relationship between each physical parameter and the tower-cable structure. This approach resulted in the creation of an integrated positioning model, which significantly enhanced the overall positioning accuracy. Although the H-DOFS has demonstrated considerable advantages, there are still areas for improvement:

1. After tower positioning, it is crucial to match the coordinates of the positioned towers with the actual geographical locations. Currently, a manual method is used for specific transmission lines, but an efficient point-matching algorithm is needed for a wider application.
2. The limitations of the current DOFS system in terms of spatial resolution and sensing distance limit the use of the amplitude difference feature for locating linear towers on longer lines. Future applications should consider this feature for shorter transmission lines to effectively incorporate tangent towers.

## 5. Conclusions

This paper proposed the integration of DOFS technology with dark fibers in OPGW to offer four potential solutions for tower location. Additionally, the introduction of H-DOFS addressed the limitations of single-parameter monitoring. This paper presents the results of tests conducted on a 220 KV line in the Fujian Province. The tests demonstrated that the method proposed in this paper has the capacity to locate 80% of the tension towers within 40 h, and it resulted in a significant increase in the number and types of positioning towers compared to the traditional method.

## Figures and Tables

**Table 1:** Parameters of the H-DOFS system.

Parameters	Value	Unit
Sensing range	46	km
Brillouin sweep step	5	MHz
Single measurement time	0.6–2	hour
Pulse width	200	ns
Repetition rate	1.8	kHz

**Table 2:** Type and number of towers for single-parameter mapping.

Tower Mapping Characteristics	Type of Towers	Numbers
BFS jump	C-type towers	12
Vibration amplitude differences	B- and C-type towers	41
Temperature differences	A-type towers	61
Strain differences	B- and C-type towers	41

**Table 3:** Comparison of tower mapping results for different mechanisms.

	BFS Jump	Vibration Amplitude Difference	Temperature Difference	Strain Difference	H-DOFS
Anchor point ratio	7.80%	39.20%	51.00%	36.30%	80.40%
Dependence on meteorological environment	No	No	Yes	Yes	Yes
Time to achieve 80% identification of B- and C-type towers	Unachievable	54H	Unachievable	142H	40H
Interpolation to calculate positioning error	65 m	119 m	124 m	88 m	84 m

## Data Availability

The data presented in this study are available on request from the corresponding author. The data are not publicly available due to privacy.

## Abbreviations

The following abbreviations are used in this manuscript:

DOFS	distributed optical fiber sensing
OPGW	optical fiber composite overhead ground wire
BFS	Brillouin frequency shift
H-DOFS	hybrid distributed optical fiber sensing
RBS	Rayleigh backscattered signal
